# Miscellany

**Published:** 1889-04

**Authors:** 


### MISCELLANY.

***Seventh Decennial Convention for Revising the Pharmacopœia of the United States.*** —Notice is hereby given that, in accordance with and by virtue of the authority vested in me by the Convention of 1880, I hereby call upon the several incorporated Medical Societies, incorporated Medical Colleges, incorporated Colleges of Pharmacy, and incorporated Pharmaceutical Societies throughout the United States of America, The American Medical Association, and The American Pharmaceutical Association, to elect a number of delegates, not exceeding *three*, and upon the Surgeon-General of the Army, Surgeon-General of the Navy, and the Surgeon-General of the Marine Hospital Service to appoint, each, not exceeding *three* medical officers to attend a General Convention for the Revision and Publication of the Pharmacopœia of the United States of America, to assemble in the city of Washington, D. C., on the first Wednesday of May, 1890 (May 7th), at twelve o'clock noon.

The several bodies, as well as the Medical Departments of the Army, Navy and Marine Hospital Service, are hereby requested to submit the Pharmacopœia to a careful revision, and to transmit the result of their labors to the Committee of Revision at least three months before the meeting of the General Convention.

The several Medical and Pharmaceutical bodies are hereby requested to transmit to me, as the President of the Convention of 1880, the names and residences of their respective delegates, as soon as they shall have been appointed; a list of these delegates shall thereupon be published under my authority, for the information of the medical public, in the newspapers and medical journals in the month of March, 1890.

In the event of the death, resignation or inability of the President of the Convention of 1880 to act, these duties (in accordance with the Resolution of that Convention) shall devolve, successively, in the following order of precedence: upon the Vice-Presidents, the Secretary, the Asst. Secretary, and the Chairman of the Committee of Revision and Publication of the Pharmacopœia.

These officers are as follows: *First Vice-President*, Samuel C. Busey, M. D., of Washington, D. C.; *Second Vice-President*, P. W. Bedford, Ph. G., of New York; *Secretary*, Frederick A. Castle, M. D., of New York; *Assistant Secretary*, C. H. A. Kleinschmidt, M. D., of Washington, D. C.; *Chairman of the Committee of Revision*, Charles Rice, Ph. D., of New York; *First Vice-Chairman of the Committee of Revision*, Joseph P. Remington, Ph. M., of Philadelphia, Pa.; *Second Vice-Chairman of the Committee of Revision*, C. Lewis Diehl, Ph. G., of Louisville, Ky.

At the General Convention held in Washington, D. C., on the fifth day of May, 1880, the organizations and bodies enumerated in the Abstract of the Proceedings of the National Convention of 1880, on pp. xv. to xviii. of the U. S. Pharmacopœia of 1882, were recognized as being entitled to representation.

If any body other than those admitted in 1880 shall desire a representation in the Convention of 1890, it is suggested that the proof of incorporation, signed by the Secretary of State, of the State which shall have issued the charter, or by properly qualified public officials of the United States, be presented with the credentials of the delegation.

A blank form of certificate of appointment of delegates will be sent upon application by letter to my address, care of Dr. Edwin H. Brigham, Assistant Librarian of the Boston Medical Library, 19 Boylston Place, Boston, Mass.

(Signed) ROBERT AMORY,
*President of the Convention of 1880.*
Boston, March 9th, 1889.

---

***The Death of Professor Donders,*** the celebrated Professor of Physiology in the University of Utrecht, the latter part of March, removes a man whose name and labors were known throughout the civilized world. He was born at Tilburg in 1818. In 1848 he took up the special study of the then new science of ophthalmology, and in 1851 founded the first hospital for the treatment of diseases of the eye in Holland. Since that time he has published a great deal on the diseases of the eye. His resignation from his chair last year was made the occasion of a ceremonial, when $15,000 was presented to him by his pupils and admirers. This sum he generously devoted to the establishment of scholarships in physiological and ophthalmological science.

---

*The Illinois State Medical Society* will hold its next annual meeting in Jacksonville on the third Tuesday in May. It will be an important meeting in many respects, and there should be a full attendance of the members.

Dr. T. M. Cullimore, Assistant Secretary, furnishes the following list of railroads which have agreed to sell tickets for one-third fare in returning, to those who paid full fare in going to the meeting:

Chicago and Alton Railway.

Chicago and Northwestern Railway.

Chicago, Burlington and Northern Railway.

Chicago, Burlington and Quincy Railway.

Chicago, Milwaukee and St. Paul Railway.

Chicago, Rock Island and Pacific Railway.

Chicago, St. Paul and Kansas Railway.

Chicago, Santa Fe and California Railway.

Illinois Central Railway.

Rock Island and Peoria Railway.

Wisconsin Central Lines Railway.

Wabash Railway.

Jacksonville Southeastern Railway.

---

*Bacteria and Books.*—A good deal of discussion has been going on as to whether books may convey pathogenic bacteria. Recent researches at Dresden tend to show that pathogenic bacilli are not capable of living in or on books for any length of time. The details of the experiments have not been published. Meanwhile, it is probably best to assume that books may be "inoculated" by persons suffering with infectious diseases.

---

*The Annual Meeting of the National Association of Railway Surgeons* will be held at St. Louis, Mo., on Thursday and Friday, May the 2d and 3d, 1889. The prospects are that this will be one among the largest gatherings of medical men ever assembled in this country. Dr. W. B. Outten, of St. Louis, is the Chairman of the Committee of Arrangements, and everything will be complete for the accommodation of the surgeons. Any information desired can be had by addressing the Secretary, C. B. Stemen, M.D., Fort Wayne, Ind.

---

*The American Medical Association* will hold its fortieth annual meeting at Newport, R. I., on June 25, 26, 27, and 28, 1889. From reports already published by the Secretaries of the Sections, the meeting promises to be one of unusual interest. The general addresses will be delivered by Professors William Pepper of Philadelphi, Wm. H. Welch of Baltimore, and P. S. Conner of Cincinnati.

---

*The Death of Dr. C. J. B. Williams,* at the age of 84, is announced. He retired from practice in 1875, but previous to that time was one of the best known and most eminent of British physicians. He may be regarded as the one that really introduced cod-liver oil as a means of treating consumption.

---

*An International Congress of Otology and Laryngology* is to be held in Paris, France, during the International Exposition of this year. The date of the proposed Congress is from the 16th to 21st of September. The Trocadéro palace is to be the place of meeting.

